# A single-center experience on long-term clinical performance of a rapid SARS-CoV-2 Antigen Detection Test, STANDARD Q COVID-19 Ag Test

**DOI:** 10.1038/s41598-023-48194-2

**Published:** 2023-11-27

**Authors:** Seo Wan Kim, Yongjung Park, Dokyun Kim, Seok Hoon Jeong

**Affiliations:** 1 Department of Laboratory Medicine, Gangnam Severance Hospital, Yonsei University College of Medicine, 211 Eonju-ro, Gangnam-gu, Seoul, 06273 South Korea; 2 Research Institute of Bacterial Resistance, Yonsei University College of Medicine, Seoul, South Korea

**Keywords:** Infectious diseases, Infection

## Abstract

The COVID-19 pandemic in Korea has dynamically changed with the occurrence of more easily transmissible variants. A rapid and reliable diagnostic tool for detection of SARS-CoV-2 is needed. While RT-PCR is currently the gold standard for detecting SARS-CoV-2, the procedure is time-consuming and requires expert technicians. The rapid antigen detection test (RADT) was approved as a confirmatory test on 14 March 2022 due to rapid dissemination of the Omicron variant. The benefits of the RADT are speed, simplicity, and point-of-care feasibility. The aim of our study was to evaluate the clinical performance of RADT compared to RT-PCR in a single center over 15 months, fully covering the SARS-CoV-2 ‘Variants of Concern (VOC).’ A total of 14,194 cases was simultaneously tested by RT-PCR and RADT from January 2021 to March 2022 in Gangnam Severance Hospital and were retrospectively reviewed. PowerChek SARS-CoV-2, Influenza A&B Multiplex Real-time PCR Kit, and STANDARD Q COVID-19 Ag Test were used. Positive rates, sensitivities, specificities, positive predictive values (PPV), and negative predictive values (NPV) were estimated for five periods (3 months/period). Receiver operator characteristic curve (ROC) analysis was performed, and Spearman’s rank test assessed the correlation between RT-PCR Ct values and semi-quantitative RADT results. The overall positive rate of RT-PCR was 4.64%. The overall sensitivity and specificity were 0.577 [95% confidence interval (CI) 0.539–0.614] and 0.991 [95% CI 0.989–0.993], respectively. ROC analysis resulted in an area under the curve of 0.786 (*P* < 0.0001, Yuden’s index = 0.568). The PCR positive rates were estimated as 0.11%, 0.71%, 4.51%, 2.02%, and 13.72%, and PPV was estimated as 0.045, 0.421, 0.951, 0.720, and 0.798 in Periods 1, 2, 3, 4, and 5, respectively. A significant and moderate negative correlation between PCR Ct values and semi-quantitative RADT results was observed (Spearman’s ρ = − 0.646, *P* < 0.0001). The RADT exhibited good performance in specimens with low Ct values (Ct ≤ 25.00) by RT-PCR. The PPV was significantly higher in Periods 3 and 5, which corresponds to rapid dissemination of the Delta and Omicron variants. The high PPV implies that individuals with a positive RADT result are very likely infected with SARS-CoV-2 and would require prompt quarantine rather than additional RT-PCR testing. The sensitivity of 0.577 indicates that RADT should not replace RT-PCR. Nonetheless, given the high PPV and the ability to track infected persons through rapid results, our findings suggest that RADT could play a significant role in control strategies for further SARS-CoV-2 variants.

## Introduction

The World Health Organization declared a global pandemic of severe acute respiratory syndrome coronavirus 2 (SARS-CoV-2) infection, a novel coronavirus disease 2019 (COVID-19), in March 2020. In accordance with global trends, the national situation of COVID-19 in Korea has changed dynamically. The Delta variant was disseminated in early August 2021, and the Omicron variant replaced the Delta variant in December 2021^1–4^**.** The number of COVID-19 cases escalated in each event, with the appearance of variants of higher transmissibility; therefore, it is necessary to prepare a rapid and reliable diagnostic tool for detection of SARS-CoV-2. Real-time reverse transcription PCR (RT-PCR) has been the gold standard in detecting SARS-CoV-2 due to its high sensitivity and specificity^5,6^. Nevertheless, it is time-consuming and requires expert technicians to be performed accurately^7–9^. Rapid antigen detection tests could be an alternative option for diagnosis of COVID-19 infection.

Initial approval of a rapid SARS-CoV-2 Antigen Detection Test (RADT) was granted in Korea in November 2020. The RADT was approved for use in areas with urgent needs including emergency rooms, intensive care units, and long-term care facilities. The RADT exhibits lower sensitivity in detecting COVID-19 patients compared with PCR-based methods; however, the advantages of RADT are rapidity, simplicity, and point-of-care feasibility with a 15–20 min turnaround time^10^. There have been several studies on the diagnostic performance of RADTs compared with results obtained by RT-PCR; however, most of them were performed with small numbers of samples or were analyzed with inconsistent sample types, reagents, and test kits. Therefore, the clinical performance of RADTs has been reported in a variety of ranges with heterogeneity. In addition, there was a limitation in those prior studies that variants of SARS-CoV-2 could not be fully evaluated^11–14^. The aim of our study was to evaluate the clinical performance of the STANDARD Q COVID-19 Ag Test (SD Biosensor Inc., Gyeonggi-do, Korea) compared to RT-PCR with a large number of samples over a long period, covering the Alpha, Delta, and Omicron variant pandemics. We also analyzed the diagnostic performance of the STANDARD Q COVID-19 Ag Test according to the change of positive rates of SARS-CoV-2**.**

## Materials and methods

### Study population

**Figure 1:**
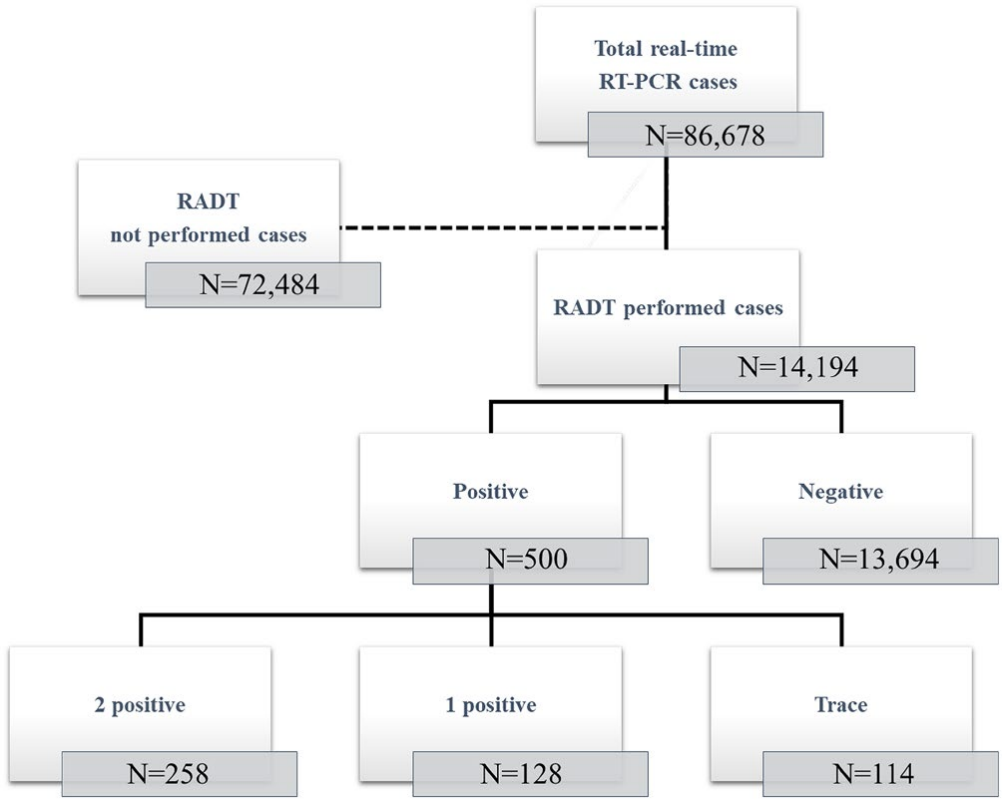
Results of real-time PCR and rapid antigen detection test of SARS-CoV-2 during the study period.

To evaluate the clinical performance of RADT comparing with the results of RT-PCR, a retrospective chart review was performed. Inclusion criteria was the cases subjected for detection of SARS-CoV-2 by both RT-PCR and RADT in same day from January 2021 to March 2022. A total of 86,678 samples was tested for detection of SARS-CoV-2 by RT-PCR, and 14,194 cases simultaneously tested by RADT were included in this study (Fig. 1). The nasopharyngeal swab (NPS) and oropharyngeal swab (OPS) of the patients were collected and transported via viral transport medium (VTM) (AB Transport Medium (AB MEDICAL, Seoul, Korea)) and were used in performing RT-PCR and RADTs to detect SARS-CoV-2. All processes of this study were in accordance with the ethical standards of the institutional and national research committee and with the 1964 Helsinki declaration and comparable ethical standards. This study was approved by the Institutional Review Board of Yonsei University Gangnam Severance Hospital (approval number: 3-2023-0243) to waive the requirement for informed consents about the collection of clinical data.

### SARS-CoV-2 RNA detection using RT-PCR

RNA was extracted from 300 $\mathrm{\mu L}$ of VTM using the TANBead Nucleic Acid Extraction Kit (Taiwan Advanced Nanotech Inc., Taiwan) according to the manufacturer’s instructions. RT-PCR of SARS-CoV-2 was performed with PowerChek SARS-CoV-2, Influenza A&B Multiplex Real-time PCR Kit (KOGENEBIOTECH Co., Seoul, Korea). The PowerChek assay simultaneously targets the envelope (*E*) and open reading frame 1ab (*ORF1ab*) genes of SARS-CoV-2. One drop of 5 μL of extracted template RNA was added to 15 μL of PCR reaction mixture (2X RT-PCR Master Mix 10 $\mathrm{\mu L}$ & Primer/Probe Mix 5 $\mathrm{\mu L}$). A total volume of 20 μL was loaded and amplified 40 cycles by the CFX96 Real-time PCR Detection System (Bio-Rad Laboratories, Inc., Hercules, CA). Exponential fluorescence curves that crossed the threshold line at or before 38 cycles (cycle threshold [Ct] ≤ 38) for both the SARS-CoV-2 *E* gene and *ORF1ab* gene were considered a positive result. A negative result was determined when Ct values for both genes were above 40 (Ct > 40). The indeterminate results were defined when Ct value of only one of the *E* gene and the *ORF1ab* gene was below 38 and when Ct values of either *E* gene or *ORF1ab* gene was between 38 and 40.

### Rapid SARS-CoV-2 Antigen Detection Test (RADT)

The STANDARD Q COVID-19 Ag Test was approved by the MFDS of Korea in November 2020. This test is a lateral flow immunochromatographic assay (ICA) that detects the nucleocapsid protein (NP) antigen of SARS-CoV-2 semi-quantitatively. It has two pre-coated lines on the nitrocellulose membrane: control (C) and test (T) lines. A sample of 350 μL of VTM was mixed with the extraction buffer solution, and 2–3 drops of the sample mixture were applied on the sample area of the device. After 15–20 min of incubation at room temperature, results were determined manually with the naked eye. For a positive result, two colored C and T lines were present within 30 min. The test was interpreted as ‘trace’ if the T line was fainter than the C line; ‘1+ (one positive)’ if the T line showed similar intensity to the C line; and ‘2+ (two positive)’ if the T line was thicker than the C line. For a negative result, only the C line was present.

### Statistical analysis

Analyse-it$\circledR$ (version 5.68; Analyse-it Software Ltd., Leeds, UK) was used for all statistical analyses. Differences between groups were analyzed using Kruskal–Wallis tests for continuous variables and Chi-square tests for categorical variables. Spearman’s rank test was performed to assess the correlation between results by RT-PCR and RADT. Sensitivity, specificity, positive predictive value (PPV), and negative predictive value (NPV) were calculated for the results obtained by the STANDARD Q COVID-19 Ag Test compared with those by RT-PCR. The 95% confidence intervals (CIs) were estimated by Wilcoxon sum rank test. To evaluate the diagnostic performance of the STANDARD Q COVID-19 Ag Test, receiver operating characteristics (ROC) curve analysis was performed, and the area under curve (AUC) was estimated. Statistical significance was determined when the *P* value was less than 0.05.

## Results

### Characteristics of cases, PCR results, and *ORF1ab* gene Ct values by period

Among the 14,194 cases, 500 positive and 13,694 negative results were obtained. The positive results consisted of 258 results of 2+, 128 results of 1+, and 114 results of trace (Fig. 1).

The evaluation period was divided into 5 periods (3 months per period), and the number of cases examined using the STANDARD Q COVID-19 Ag Test by period ranged from 1822 to 3217. The gender ratio, median age, PCR qualitative results, and *ORF1ab* gene Ct value distribution are shown in Table 1. The PCR positive rate was estimated to be 0.11% (2/1822) in Period 1, surged to 4.51% (145/3217) in Period 3 (the Delta variant epidemic), and peaked at 13.72% (429/3127) in Period 5 (the Omicron variant epidemic). The proportion of cases with Ct values less than 25.00, indicating a high viral load, was 0.05% in Period 1, increased to 2.55% in Period 3, and peaked at 6.78% in Period 5.

**Table 1:** Summary of SARS-CoV-2 rapid antigen detection test results.

Variables	Total (Jan 2021–Mar 2022) N = 14,194	Period 1 (Jan 2021–Mar 2021) N = 1822	Period 2 (Apr 2021–Jun 2021) N = 2958	Period 3 (Jul 2021–Sep 2021) N = 3217	Period 4 (Oct 2021–Dec 2021) N = 3070	Period 5 (Jan 2022–Mar 2022) N = 3127	*P* value
Female, N (%)	7,062 (49.75)	820 (45.01)	1,467 (49.59)	1,692 (52.60)	1,506 (49.06)	1,577 (50.43)	< 0.0001
Age (years) (Median, 1st to 3rd quartiles)	53.0 (30.0–71.0)	60.0 (40.0–73.0)	54.0 (32.0–72.0)	52.0 (30.0–70.0)	51.0 (28.0–70.0)	49.0 (28.0–68.0)	< 0.0001
Symptomatic case, N (%)	7,355 (51.8)	397 (21.8)	1,529 (51.7)	1,906 (59.2)	1,732 (56.4)	1,791 (57.3)	< 0.0001
Onset duration of symptomatic patients (days) (Median, 1st to 3rd quartiles)	1 (0.0 – 3.0)	0.5 (0.0 – 2.5)	1 (0.0 – 3.0)	1.0 (0.0 – 3.0)	1.0 (0.0 – 2.8)	1.0 (0.0 – 3.0)	0.2805
PCR results, N (%)							
Positive	659 (4.64)	2 (0.11)	21 (0.71)	145 (4.51)	62 (2.02)	429 (13.72)	< 0.0001
Indeterminate	51 (0.36)	0 (0.00)	1 (0.03)	5 (0.15)	5 (0.16)	40 (1.28)	< 0.0001
Negative	13,484 (95.00)	1,820 (99.89)	2,936 (99.26)	3,067 (95.34)	3,003 (97.82)	2,658 (85.00)	< 0.0001
ORF1ab gene Ct value, N (%)							
< 20.00	157 (1.11)	1 (0.05)	9 (0.30)	43 (1.34)	14 (0.46)	90 (2.88)	< 0.0001
20.00–24.99	191 (1.35)	0 (0.00)	5 (0.17)	39 (1.21)	25 (0.81)	122 (3.90)	< 0.0001
25.00–29.99	132 (0.93)	0 (0.00)	2 (0.07)	24 (0.74)	10 (0.33)	96 (3.07)	< 0.0001
30.00–39.99	219 (1.54)	1 (0.05)	6 (0.20)	43 (1.34)	17 (0.55)	152 (4.86)	< 0.0001
≥ 40.00	13,495 (95.07)	1,820 (99.90)	2,936 (99.26)	3,068 (95.37)	3,004 (97.85)	2,667 (85.29)	< 0.0001

### Correlation between Ct value of *ORF1ab* gene PCR and semiquantitative results obtained by STANDARD Q COVID-19 Ag Test

**Figure 2:**
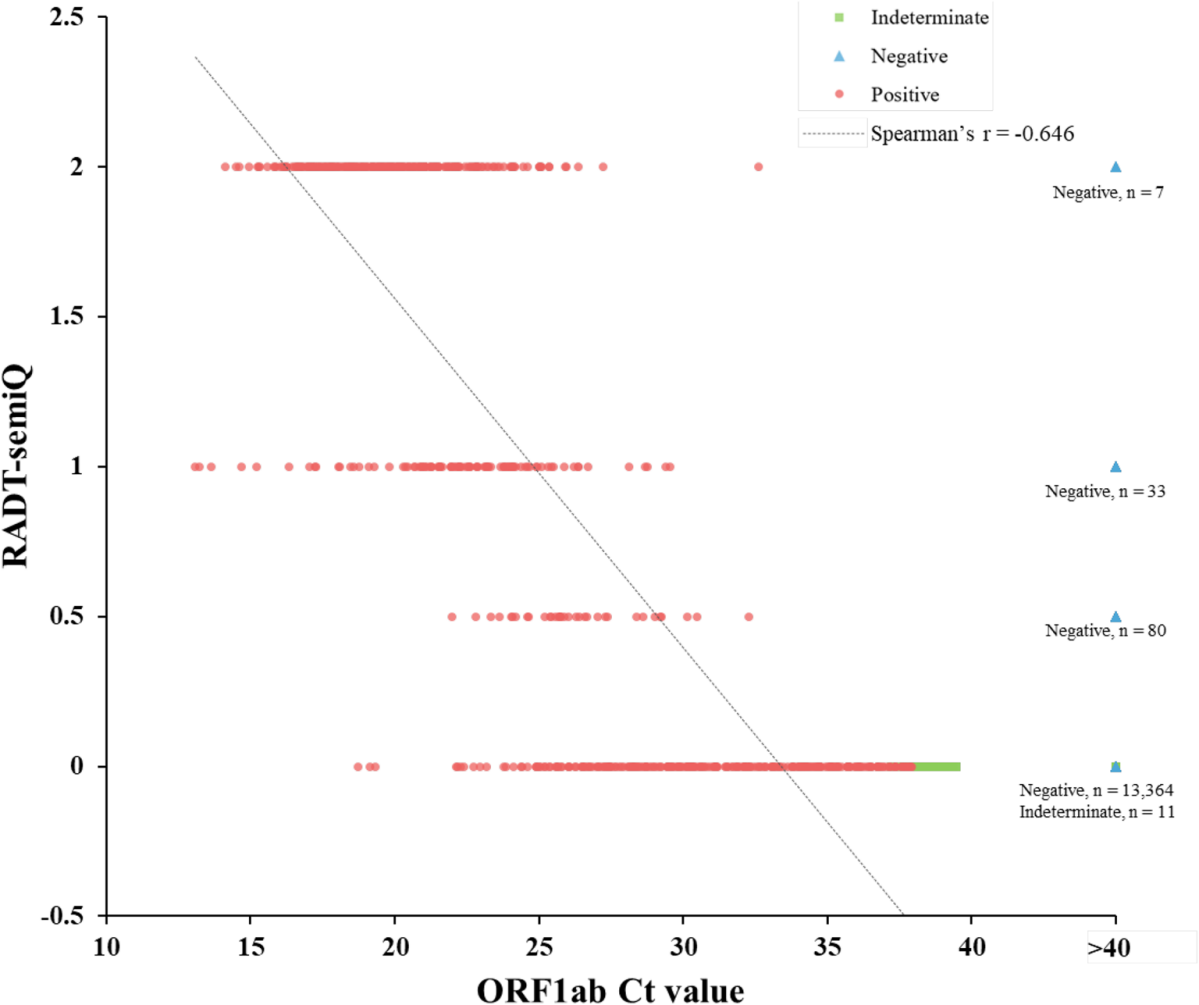
Correlation between Ct value (ORF 1ab gene) of real-time RT-PCR and RADT semi-quantitative results, including indeterminate results. The number of overlapping negative or indeterminate case dots with Ct value of ORF1ab over 40 were added in the graph.

Table 2 summarizes the qualitative and semiquantitative results of the STANDARD Q COVID-19 Ag Test according to the Ct values of *ORF1ab* gene PCR, including indeterminate results. Most (329/348, 94.5%) of the cases with low CT values under 25.00 gave a positive result with the STANDARD Q COVID-19 Ag Test. In addition, most (137/157, 89.0%) of the cases with Ct values under 20.00 showed 2+, and more than half (102/191, 58.3) of the cases with Ct values of 20.00–24.99 also showed 2+ with the STANDARD Q COVID-19 Ag Test. When semiquantitative results of the STANDARD Q COVID-19 Ag Test were compared with Ct values obtained by RT-PCR, a significant and moderate negative correlation was observed (Spearman’s ρ = − 0.646, *P* < 0.0001) (Fig. 2).

**Table 2:** Correlation between RADT and ORF1ab gene Ct values of real-time RT-PCR including indeterminate results.

RADT results		Real-time RT-PCR: *ORF 1ab* gene Ct value				
		< 20.00 N = 157	20.00–24.99 N = 191	25.00–29.99 N = 132	30.00–39.99 N = 219	≥ 40.00 N = 13,495
Qualitative, N (%)	Positive	154 (98.09)	175 (91.62)	47 (35.61)	4 (1.83)	120 (0.89)
	Negative	3 (1.91)	16 (8.38)	85 (64.39)	215 (98.17)	13,375 (99.11)
Semi-quantitative, N (%)	2+	137 (88.96)	102 (58.29)	11 (23.40)	1 (25.00)	7 (5.83)
	1+	17 (11.04)	64 (36.57)	14 (29.79)	0 (0.00)	33 (27.50)
	Trace	0 (0.00)	9 (5.14)	22 (46.81)	3 (75.00)	80 (66.67)
	Negative	3 (1.91)	16 (8.38)	85 (64.39)	215 (98.17)	13,375 (99.11)

### Clinical performance of the RADT

**Figure 3:**
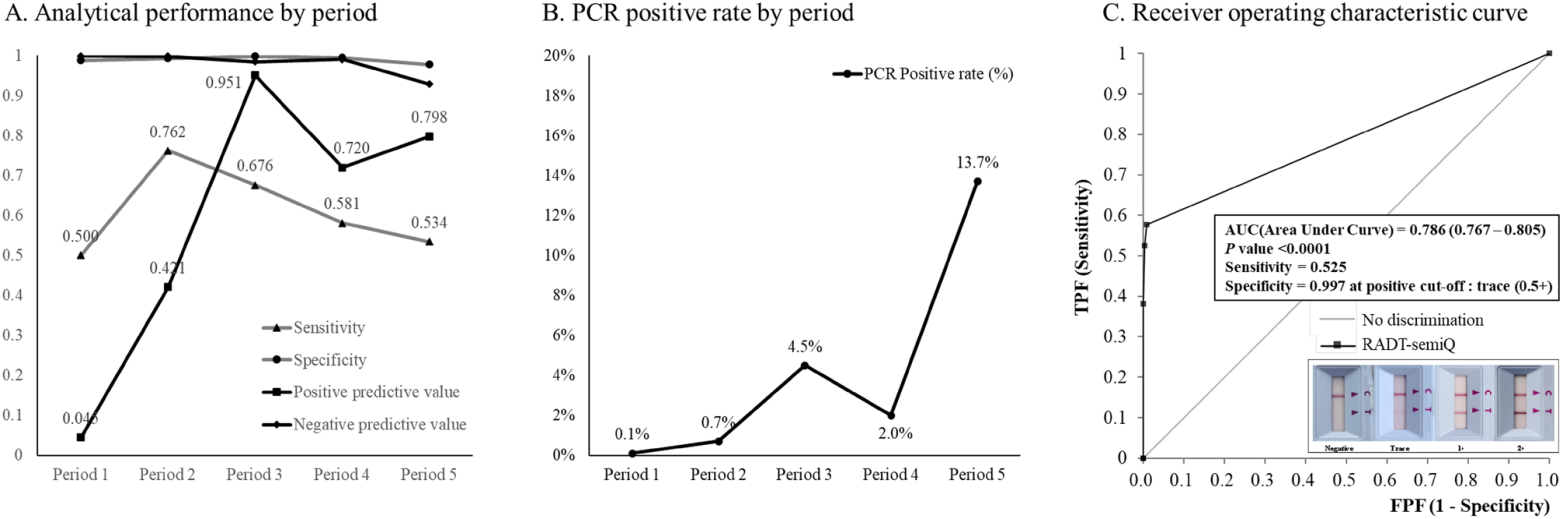
PCR positive rate and analytical performance by period and receiver operating characteristics curve of RADT. (**A**) Sensitivity, specificity, positive predictive value, and negative predictive value by period, (**B**) PCR positive rate by period, and (**C**) receiver operating characteristic curve of RADT.

The PCR positive rates, clinical performance of the STANDARD Q COVID-19 Ag Test compared with the PCR results, and PPV and NPV by period are summarized in Table 3. The overall sensitivity, specificity, PPV, and NPV were 0.577 (95% CI 0.539–0.614), 0.991 (95% CI 0.989–0.993), 0.760 (0.724–0.795), and 0.980 (95% CI 0.978–0.981), respectively. The PPV was lowest in Period 1 (0.045; 95% CI 0.011–0.169) and high in Period 3 (0.951; 95% CI 0.890–0.979) and Period 5 (0.798; 95% CI 0.751–0.838). The specificity was greater than 0.970 in all periods. ROC curve analysis resulted in an AUC of 0.786 (*P* < 0.0001) and Youden’s index of 0.568 (Fig. 3). The monthly trends of sensitivity, specificity, PPV, NPV, and PCR positive rates are described in Supplementary Figure S1**.**

**Table 3:** Diagnostic performance of RADT compared to real-time RT-PCR (except indeterminate results).

Parameters	Period 1 (Jan 2021–Mar 2021) N = 1822	Period 2 (Apr 2021–Jun 2021) N = 2957	Period 3 (Jul 2021–Sep 2021) N = 3212	Period 4 (Oct 2021–Dec 2021) N = 3065	Period 5 (Jan 2022–Mar 2022) N = 3087	Total (Jan 2021–Mar 2022) N = 14,143
Sensitivity	0.500 (0.095–0.905)	0.762 (0.549–0.894)	0.676 (0.596–0.747)	0.581 (0.457–0.695)	0.534 (0.487–0.580)	0.577 (0.539–0.614)
Specificity	0.988 (0.982–0.992)	0.993 (0.989–0.995)	0.998 (0.996–0.999)	0.995 (0.992–0.997)	0.978 (0.972–0.983)	0.991 (0.989–0.993)
Positive predictive value	0.045 (0.011–0.169)	0.421 (0.310–0.540)	0.951 (0.890–0.979)	0.720 (0.594–0.819)	0.798 (0.751–0.838)	0.760 (0.724–0.793)
Negative predictive value	0.999 (0.998–1.000)	0.998 (0.996–0.999)	0.985 (0.981–0.988)	0.991 (0.988–0.994)	0.929 (0.922–0.935)	0.980 (0.978–0.981)
PCR positive rate (%)	0.11	0.71	4.51	2.02	13.72	4.64

## Discussion

Antigen-based immunoassays for detecting SARS-CoV-2 were introduced for rapid diagnosis of COVID-19. In this study, the clinical performance of the STANDARD Q COVID-19 Ag Test, an immunochromatographic assay, was evaluated in comparison with results obtained by the RT-PCR method.

There have been previous studies evaluating the clinical performance of RADTs for diagnosing COVID-19 infection. In a German study, 2,028 samples collected from a variable population including patients with COVID-19 symptoms, individuals with SARS-CoV-2 exposure, and hospital staff members showed overall sensitivity and specificity of 42.86 and 99.89%, respectively^15^. In a study from Serbia, 120 symptomatic patients were tested by the RADT, and overall sensitivity was about 60%^16^. In another study from Egypt performed with COVID-19 suspected individuals, the sensitivity and specificity were 78.2% and 64.2%, respectively^17^. A previous study conducted in Korea with pre-hospital patients, emergency room visitors, and patients confirmed with COVID-19 by RT-PCR demonstrated an overall sensitivity of 17.5% and a specificity of 100%^18^. This study was performed with a large number of clinical samples over 15 months, provided more reliable results reflecting practical aspects compared with other evaluation studies, and minimized bias that might occur in selection of study target samples. Overall sensitivity and specificity of the RADT were 0.577 (95% CI, 0.539–0.614) and 0.991 (95% CI, 0.989–0.993) respectively, which are comparable to the results reported in previous studies^16–18^. In addition, three cases of false-negative results in the cases with Ct value < 20. Clerical errors, inappropriate specimen handling, or interference in specimens could be potential reasons^19^. A total 120 cases (0.89%) of false-positive results were identified in this study. According to the previous report in Canada, the false positive results were identified in 0.05% (462/903,408) of asymptomatic screening tests^20^, and a false positive rate in another study in Germany was reported to be 0.13% (1561/1,245,962)^21^. False positive results may lead to unnecessary quarantine and delay of appropriate procedure to patients. The AUC of RADT was suboptimal (0.781) due to relatively low sensitivity and confirmation test with RT-PCR assay should be performed for accurate diagnosis of SARS-CoV-2 infection.

Significant correlation was identified between the Ct values obtained by RT-PCR and semiquantitative results by RADT, and the accuracy of RADT was very high, particularly in cases with high viral loads, exhibiting low Ct values^22,23^. In the German study, higher sensitivities were noted as Ct values < 20, < 25, and < 30 produced results of 100%, 98.25%, and 88.64%, respectively^15^. Therefore, the RADT could be accurate in diagnosing SARS-CoV-2 infection in the early stage, in which a high viral load is usually identified.

PPV and NPV are the most important indexes to determine clinical relevance and could vary according to disease prevalence. During the study period, steep increases in prevalence were observed in Periods 3 and 5, which correspond to the Delta and Omicron variant epidemics, respectively. As the prevalence increased, PPVs were confirmed to increase in Period 3 (0.951). The high PPV in the COVID-19 epidemic indicates that an individual with a positive RADT result is highly suspected to be infected with SARS-CoV-2 and should be required to promptly quarantine rather than undergo additional RT-PCR testing. Meanwhile, the PCR positive rate in Period 5 (13.7%) was higher than that in Period 3 (4.5%), but PPV in Period 5 was lower than that in Period 3. During the Period 5, the number of positive cases were dramatically increased up to 4.1% (55/1336), and Ct value of more than half of these cases were ≥ 25.00.

Most RADTs target the nucleocapsid protein stably associated with RNA in the virion and is found at higher level than other viral structural proteins^24^. Prior to the introduction of VOCs, the majority of RADTs was developed with strains that lacked nucleocapsid mutations^25^, and there have only been a few clinical validation studies that have examined variants affecting antigen test performance. From our results, it can be inferred that the Omicron and Delta variants, which are characterized by many mutations in the spike protein, had little effect on the accuracy of the nucleocapsid targeting RADT. Nonetheless, Omicron and Delta variants also harbor nucleocapsid proteins involving mutations (i.e., Omicron; P13L, ∆31–33, R203K & Delta; D63G, R203M, D377Y), in conjunction with the spike protein's conspicuous alterations^26,27^. While there are some false-negative reports of specimens carrying nucleocapsid mutations that show a high viral load as confirmed by PCR^28,29^, it is considered not to affect the accuracy of the RADT since current tests target the C-terminus of the nucleocapsid protein, whereas the great majority of mutations is identified in the N-terminus. In our study, no substantial shift or inclination in specificity was identified according to variant, which indicates the variants did not affect the clinical performance of the RADT. However, we must remain cautious to detect changes in the sensitivity of RADT due to variations with specific mutation trends, and additional studies are necessary to validate this point.

One of the limitations of this study is that the samples were collected in a single center in a single country; therefore, ethnic or racial diversities could not be considered. In addition, samples tested for pre-hospitalization screening were included; therefore, some asymptomatic patients were inevitably included in our study, which would lower the positive rate. The lack of further investigation about false-positive and false-negative results due to the retrospective study design was another limitation of this study. Finally, STADNARD Q COVID-19 Ag was approved for testing the detection of SARS-CoV-2 by Ministry of Food and Drug Safety of the Republic of Korea in both specimens including nasopharyngeal swab and VTM. Even though the limit of detection of this diagnostic kit in both specimens were validated, possible dilution effect could be occurred when using VTM as a specimen.

In conclusion, the STANDARD Q COVID-19 Ag Test showed lower sensitivity compared with RT-PCR; however, this RADT kit showed high PPV in the pandemic situation with a high prevalence of COVID-19 infection, which suggests this RADT kit could be an alternative option for rapid detection of SARS-CoV-2 in a pandemic situation. Further investigation should be performed to compare the results of other RADTs to figure out the clinical usefulness of RADT in diagnosing SARS-CoV-2 infection.

### Supplementary Information

Supplementary Information.

## Data Availability

The datasets used and/or analyzed during the current study available from the corresponding author on reasonable request.
