# Supplementary material for: A single-center experience on long-term clinical performance of a rapid SARS-CoV-2 Antigen Detection Test, STANDARD Q COVID-19 Ag Test
Source: Sci Rep. 2023 Nov 27;13:20777. doi: 10.1038/s41598-023-48194-2 (PMC10681986; doi:10.1038/s41598-023-48194-2)
Supplement: Supplementary file 1 — Supplementary Information. [file 41598_2023_48194_MOESM1_ESM.pptx]

## Slide 1
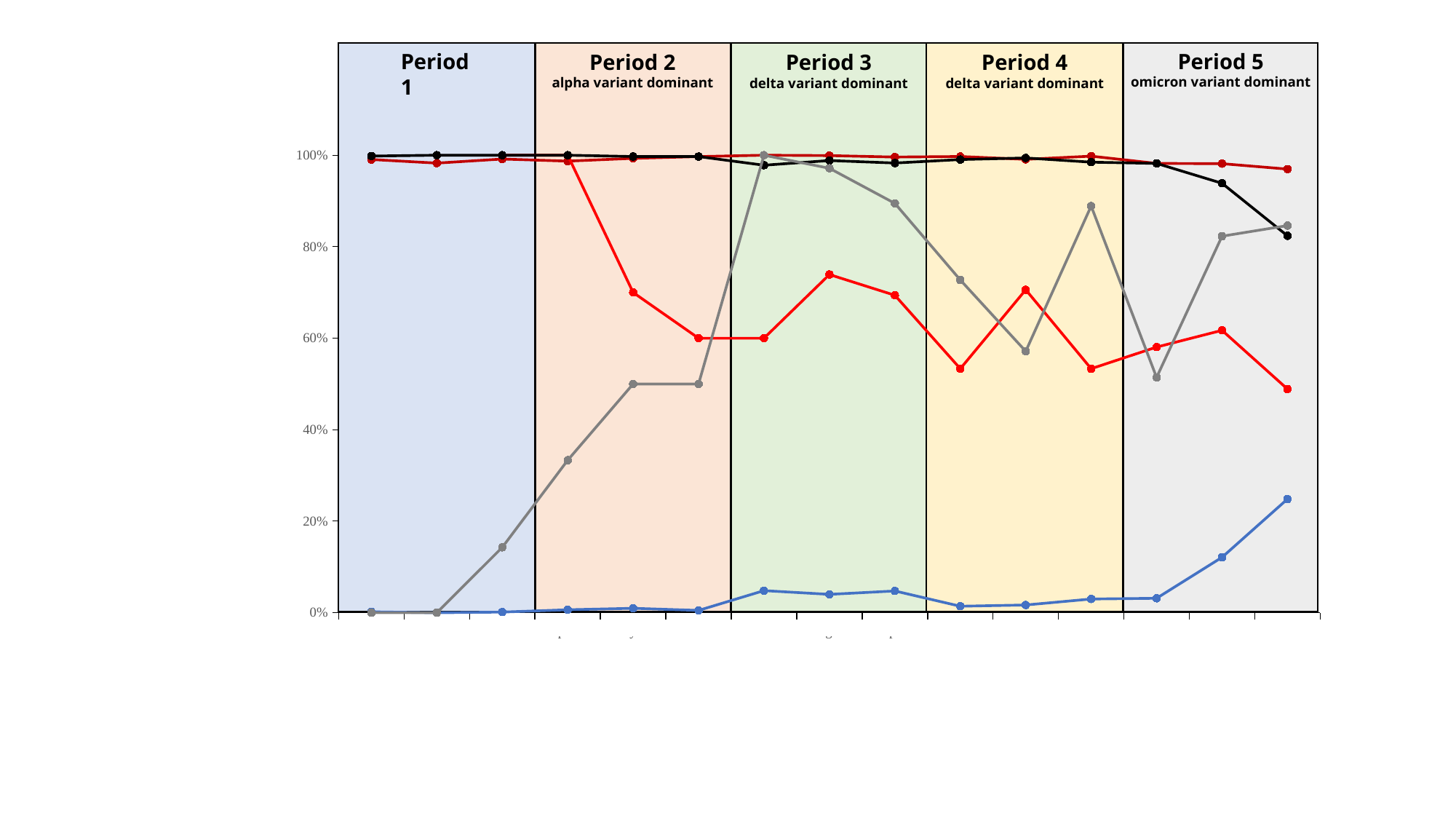

Period 5
omicron variant dominant
Period 1
Period 2
alpha variant dominant
Period 3
delta variant dominant
Period 4
delta variant dominant
### Chart
| Category | PCR positive rate (%) | Specificity | Sensitivity | Negative predictive value | Positive predictive value |
|---|---|---|---|---|---|
| 44197 | 0.001886792452830189 | 0.9905482041587902 | None | 0.9980952380952381 | 0.0 |
| 44228 | 0.0 | 0.9826989619377162 | None | 1.0 | 0.0 |
| 44256 | 0.0014005602240896359 | 0.9915848527349228 | 1.0 | 1.0 | 0.14285714285714285 |
| 44287 | 0.0064724919093851144 | 0.9869706840390879 | 1.0 | 1.0 | 0.3333333333333333 |
| 44317 | 0.009671179883945842 | 0.9931640625 | 0.7 | 0.9970588235294118 | 0.5 |
| 44348 | 0.0050150451354062184 | 0.9969727547931383 | 0.6 | 0.9969727547931383 | 0.5 |
| 44378 | 0.048262548262548256 | 1.0 | 0.6 | 0.9781312127236581 | 1.0 |
| 44409 | 0.040034812880765894 | 0.9990925589836661 | 0.7391304347826086 | 0.9883303411131059 | 0.9714285714285714 |
| 44440 | 0.04748062015503876 | 0.9959225280326198 | 0.6938775510204082 | 0.9828973843058351 | 0.8947368421052632 |
| 44470 | 0.014150943396226417 | 0.9971209213051824 | 0.5333333333333333 | 0.990467111534795 | 0.7272727272727273 |
| 44501 | 0.01684836471754212 | 0.9909182643794148 | 0.7058823529411765 | 0.9939271255060729 | 0.5714285714285714 |
| 44531 | 0.029970029970029972 | 0.9979381443298969 | 0.5333333333333333 | 0.9847405900305188 | 0.8888888888888888 |
| 44562 | 0.03153611393692777 | 0.9820675105485233 | 0.5806451612903226 | 0.9820675105485233 | 0.5142857142857142 |
| 44593 | 0.12109744560075686 | 0.9815016322089227 | 0.6171875 | 0.9386056191467221 | 0.8229166666666666 |
| 44621 | 0.24839006439742412 | 0.9696586599241467 | 0.4888888888888889 | 0.8238453276047261 | 0.8461538461538461 |
